# ICT multimedia learning affordances: role and impact on ESL learners' writing accuracy development

**DOI:** 10.1016/j.heliyon.2021.e07517

**Published:** 2021-07-10

**Authors:** Azzam Alobaid

**Affiliations:** Centre for Linguistics, School of Languages, Literature and Culture Studies, J. N. U., New Delhi 110067, India

**Keywords:** Multimedia learning tools and effects, Cognitive advantages, YouTube affordances, Captions & their adjustable settings, ESL writing accuracy development

## Abstract

This work examined the role and impact of Information and Communications Technology (ICT) tools such as YouTube as an open educational resource-cum-tool with respect to its affordances of captions and their adjustable settings like font size and color (i.e., enhanced captions of videos); these affordances were proposed for their multimedia learning effects for the development of ESL learners' writing accuracy. This work hypothesized that the frequent use of these affordances as a technique can create and enhance a multimedia learning environment which can in its turn help learners focus more on the (new) target language input, be more able to notice their errors and correct them by way of conscious comparisons between their own output and target language input. In effect, their L2 writing becomes more accurate over time. This work investigated the role and impact of these YouTube affordances on the development of ESL writing accuracy through exploring the learners' personal experience with and actual use of these affordances for the development of ESL writing accuracy over five months. Moreover, it sought to determine whether or not learners' L2 writing accuracy improved after the implementation of the proposed YouTube affordances for ESL writing improvement. Furthermore, it explored the correlation between the development of learners' L2 writing accuracy, if any, and the impact of these YouTube affordances on the development of ESL writing accuracy. The findings of this study revealed that the frequent use of ICT multimedia tools like YouTube with respect to its affordances of captions and their adjustable settings like font size and color (i.e., enhanced captions of videos) played a positive role that impacted the improvement of learners' English writing accuracy over five months. Also, the findings demonstrated that participants' L2 writing accuracy (as indicated by all the metrics used in this study for measuring the learners' writing accuracy progress) developed significantly after five months of the implementation of the proposed YouTube affordances of enhanced captions as a technique for the development of ESL writing. Furthermore, there was a statistically significant evidence, although ethically speaking this evidence was not conclusive as it was limited by the small sample size of this group of participants and thus the elicited and analyzed data, which suggested that there was a strong correlation between the development of learners' L2 writing accuracy and the frequent use of the proposed YouTube affordances of enhanced captions for the development of ESL writing accuracy. It was concluded that ICT tools such as YouTube with respect to its affordances of captions and their adjustable settings like font size and color (i.e., enhanced captions of videos) can be effectively used and recommended as a technique for the development of learners' L2 writing accuracy due to their positive and enhancing multimedia learning effects.

## 1 Introduction

Information and Communications Technology (ICT) represents “new multimedia technologies, including computer software, CD-ROM, the internet, television, film as well as internet-based project work, e-mail, chat, blogs, wikis, podcasts, and so forth” (Zhou, 2018, p. 22). In the field of EFL/ESL learning and teaching, ICT is the integration of various technologies of information and communication to benefit from their capacity for the creation, enhancement and optimization of better learning environments. One significant potential of ICT tools is the ability to create and enhance multimedia learning environments for EFL/ESL learning and teaching. Multimedia learning environment is a concept about displaying a combination of more than one media type such as text, image, graphic, drawing, sound, video and animations usually with the aid of technology for the purpose of enhancing understanding or memorization (Guan et al., 2018). A large body of extensive research into multimedia learning environments revealed that “considerable and positive influence on language learners' performance is attributed to the increase in the use and exposure to ICT multimedia tools which provide multimedia elements (i.e., the visual and/or spoken text, graphics and videos) for ESL/EFL language learners in the learning environments” (Alobaid, 2020). Sun and Dong (2004) stated that “information presented in text, spoken words, graphics, and video formats can be integrated to produce authentic, attractive, and multi-sensory/multimedia language learning environments for EFL learners”. For a summary of multimedia tools, technology, components and applications for education, see Abdulrahaman et al. (2020). YouTube is one of many web-based ICT multimedia tools widely known for its ever-increasing affordances in this regard.

The fact that multimedia learning environments are essential to efficient language learning/teaching in the 21st century classrooms is gaining more attention by researchers and practitioners who started using and examining the ICT multimedia tools like YouTube to benefit learners from their multiple cognitive^1^ advantages in the ESL/EFL learning environments (see Watkins and Wilkins, 2011; Krauskopf et al., 2012; Zhou et al., 2020; Olasina, 2017; Kabooha and Elyas, 2018; Alobaid, 2020). For instance, in a multimedia learning environment, researchers investigated the cognitive advantages of YouTube affordances such as the captioning (also known as same-language subtitles) for the overall development of learners' proficiency of the main language skills. More specifically in this regard, they reported advantages of using captions like a greater depth of language processing, focusing learners' attention, reinforcing the acquisition of vocabulary through multiple modalities, allowing learners to determine meaning through the unpacking of language chunks and prompting long-term content retention (see Winke et al., 2010; Kovacs and Miller, 2014; Danan, 2016; Sadiku, 2018; Alobaid, 2020). Abdulrahaman et al. (2020) stated that “the use of the computer-based techniques as an interface between students and what they are learning with suitable fonts and design can be very valuable”.

This work proposed using YouTube built-in affordances of captioning and its adjustable settings of font size and color (i.e., enhanced captions^2^) due to their beneficial multimedia learning effects/cognitive advantages for ESL learners and examined their effectiveness in creating and enhancing a multimedia learning environment for the development of learners' English writing accuracy. Such advantages are the ability to increase the learners' attention, especially to the new target language input and their errors/gaps, process more information in the working memory, increase their comprehension of the spoken language input and retain these information whenever required from the long-term memory.

The motivation behind conducting this study arises primarily from the constant need to draw and focus learners' attention to the target language input presented to them in a multimedia learning environment, especially when YouTube is used as a tool to create such a learning environment (Wiebe & Annetta, 2008); and second, the need to maximize the learners' uptake or comprehension and long-term retention of linguistic information gained from some YouTube video content whenever required for writing tasks. More than 100 empirical studies documented that captioning a video improves comprehension of, attention to, and memory for the video (Gernsbacher, 2015). Third, the target language input (in its spoken form) alone in such technology-based learning environments can be challenging or difficult for learners to comprehend (not to mention retaining it) due to the overwhelming nature of speech, i.e., especially lengthy or complex spoken text may overload working memory due to its transitory nature; this can make learning far from achievable (Kalyuga, 2012). Drigas and Pappas (2017) stated that promoting L2 learners' cognitive skills, such as working memory, attention, perception, visuospatial processing and various executive skills, plays a crucial role in the formation of the learning process. In this regard, the cognitive advantages of multimedia learning can be in accord with some of the key principles of one of the most recent theories of knowledge, emotional intelligence and cognitive development, namely 'The Consciousness-Intelligence-Knowledge Pyramid: An 8 × 8 Layer Model'. According to this model, through various intervention techniques and training of metacognition and cognitive skills, learners could improve all different types of intelligence such as verbal, mathematical, and visual-spatial, and certain cognitive skills like perception, understanding, memory in all its forms, pattern recognition and problem solving (Drigas and Pappas, 2017).

The above-mentioned learners' needs/learning difficulties and the cognitive advantages of using captions and YouTube as a multimedia learning tool in language education underlay the significance of this work. This study proposed using YouTube captions, which can be adjusted in terms of font size and color for their beneficial multimedia effects (i.e., creating and enhancing the language learning environment as implied in the Multimedia Principle and Signalling Principle of the Cognitive Theory of Multimedia Learning), for language learners while using YouTube as an ICT tool in ESL writing classes.

That said, this work posed the following primary question and sought to find an answer to it:

*What are the role and impact of ICT multimedia learning affordances on ESL learners' writing accuracy development, given that they are exposed to ICT multimedia learning tools like YouTube during their learning process?*

## 2 Literature review

Recent studies have shown a burgeoning interest in multimedia technology affordances for language education such as YouTube as an enhancing multimedia tool in this regard. This study surveyed the existing literature and found several studies that dealt with the development of ESL/EFL writing in relation to the effectiveness of the general use of YouTube as an ICT multimedia tool for enhancing the learners' writing experience. Alvarez-Marinelli et al. (2016) reported that with the use of the emerging ICT multimedia technology like YouTube, learners' receptive and productive language skills can be enhanced more effectively. Similarly, it was found that the use of YouTube as a multimedia tool can improve language learners' oral, aural and writing skills (Warschauer and Grimes, 2007; Kelsen, 2009; Malhiwsky, 2010). Strassman et al. (2010) proposed that the acquisition of 21st century skills like reading and writing for all learners can best be enhanced through the use of digital technologies, i.e., television, the Internet, and applications like YouTube. More specifically, Mayora (2009) concluded that some of the YouTube features like the written comments and the potential for learners to voice their ideas through constructing meaning via the stimulus of the videos can improve learners' writing skills through authentic interaction. In the same vein, Mayora (2009) and Barbeau (2010) found that YouTube multimedia videos can help the students increase their awareness, reduce the gap between them and the teacher, and the writing classroom becomes more conducive for learning. Pratiwi (2011) and Anggraeni (2012) found that YouTube multimedia videos assist learners to explore and organize ideas, choose the right words for the construction of sentences and paragraphs, produce grammatical sentences and use the mechanics of writing, i.e., punctuation and spelling. Olasina (2017) reported that the affordance of digital media like YouTube videos can be useful for academic writing. For instance, the ease of the YouTube facilitated sessions (i.e., through the ability to playback videos), the reduction of instructor intervention increased student autonomy and aided improved writing performance of the students in academic writing by adding new elements to the traditional learning techniques.

More recently, there has been a special interest in the role and advantages of captions within multimedia learning environments for the development of ESL/EFL skills. Winke et al. (2010) concluded that captions can be useful to language learners for a number of reasons such as the higher and deeper level of processing through focusing attention, enhancing the acquisition of vocabulary via various formats, and helping to figure out meaning via the unpacking of language chunks. In the same vein, other studies by (Kovacs and Miller, 2014; Danan, 2016; Sadiku, 2018) demonstrated the effective role of captioning to facilitate general comprehension, improve language encoding and enhance vocabulary recall and long-term retention.

However, with regard to the role of YouTube affordances such as the captions and their effects on the development of the writing skill, only one recent study was found to have examined the role and impact of captions for their multimedia effects per se to develop learners' ESL writing fluency. Alobaid (2020) found that there is a strong positive correlation between the development of learners' writing fluency and the exposure to and engagement with multi-media learning environments using ICT tools like YouTube in this regard, where text can be optionally available along with speech. He concluded that multimedia materials of ICT technology can make the formal and informal experience of learning English writing more effective and interactive as learning becomes more adjustable and retainable. It can be noticed from these studies that the researchers' main focus when studying the role and effect of captions on learning foreign/second languages was the general/listening comprehension, vocabulary acquisition and retention. However, significant aspects related to ESL writing improvement such as the accuracy dimension while using ICT multimedia tools like YouTube with respect to its affordances (i.e., enhanced captions) and impact on writing have not been sufficiently investigated and are still unknown and hence call our attention to further research. This work was largely motivated by questions related to *ESL learners' writing experience while they use YouTube enhanced captioning frequently and what effect it could have on their writing accuracy*; *whether learners' writing accuracy could be enhanced due to the frequent use of captions; what is the correlation between learners' writing accuracy development, if any, and the frequent use of enhanced captions.* These are all interesting questions which showed and addressed a significant knowledge gap in the field about the study on YouTube and writing and ultimately motivated the following research questions to be asked and addressed in this study.

RQ.1. What is the impact of the frequent use of the YouTube affordances (i.e., enhanced captions of YouTube videos) on the development of ESL learners' writing accuracy?
RQ.2. Does the writing accuracy of English learners improve after the implementation of the proposed YouTube affordances for the improvement of ESL writing accuracy?
RQ.3. What is the correlation between the learners' L2 writing accuracy improvement, if any, and the frequent use of the proposed YouTube affordances for the development of ESL writing accuracy?

Also, the null (H_0_) and alternative (H_a_) hypotheses required for hypothesis testing in this work were stated as the following:

H_0_ = the frequent use of the affordances of ICT multimedia tools like YouTube (i.e., enhanced captions of YouTube videos) has no impact on the development of learners' L2 writing accuracy.
H_a_ = the frequent use of the affordances of ICT multimedia tools like YouTube (i.e., enhanced captions of YouTube videos) has an impact on the development of learners' L2 writing accuracy.

To the best of our knowledge, no research has yet been done regarding the use of affordances of YouTube such as captions and their adjustable settings of font and colour with respect to their effectiveness and impact on ESL writing accuracy. Hence, this work took a further step in research of this area and proposed to use the affordances of YouTube captions and the adjustable settings which were employed for their multimedia learning effects/cognitive advantages to improve ESL learners' writing accuracy. Such multimedia learning effects/cognitive advantages are their potentials to increase the learners' attention especially to the new target language input and their errors/gaps, process more information in the working memory, increase their comprehension of the spoken language input and retain these information whenever required from the long-term memory. In fact, captions were used in this study to create a multimedia learning environment whereby the availability of synchronized text (i.e., captions), along with speech samples, diminishes the overwhelming nature of speech and helps the student to manage the cognitive load. This allows the brain to process more information in the working memory and retain them whenever required from the long-term memory as implied in the multimedia principle (Mayer, 2014). With regard to the adjustable settings of captions, i.e., font size and colour, these were not proposed for their aesthetics but rather for their effective usability to enhance the used captions, the overall multimedia learning environment and the learners' writing accuracy experience. Specifically, enhanced captions were proposed to draw and increase learners' attention to the new target language input and their errors/gaps and support information retention because important linguistic elements are focused on through font size and colour as implied in the signalling principle, i.e., to make the information on the display clearer and the display more attractive (Mayer, 2009).

This study focused on the development of English writing accuracy in terms of six quantifiable metrics (see the data analysis section 3.4.) widely used in the literature as indicators of writing accuracy progress.

The broad sense of accuracy (or correctness) refers “to the extent to which an L2 learner's performance (and the L2 system that underlies this performance) deviates from a norm (i.e., usually the native speaker). Such deviations from the norm are traditionally labelled 'errors'” (Wolfe-Quintero et al., 1998, as cited in Housen et al., 2012). In this work, accuracy or correctness of writing was determined with respect to learners' errors in grammar, morphology, vocabulary and mechanics of writing.

### 2.1 Framework of this study

This study falls within the Cognitive Theory of Multimedia Learning (CTML) (Mayer, 2009) and Noticing the Gap Hypothesis (Schmidt, 1990, 2001). CTML states that new knowledge can be learnt through electronic instructional materials presented both verbally and visually. The affordances of YouTube like the captioning and its adjustable settings (i.e., font size and colour) were proposed in this work to create a multimedia learning environment to help ESL learners learn and improve their English writing.

#### 2.1.1 Multimedia principle

Mayer (2014) suggested that learning can be more effective when learners are presented with words and pictures rather than words alone. Nonetheless, the term multimedia includes multiple modalities of visual (i.e., photographs, charts, pictures, graphs, illustrations, and especially animations and videos) and verbal (narrations, spoken words/sounds, and texts) representations presented together (Butcher, 2006; 2014, as cited in Kanellopoulou et al., 2019); learning can be aided as learners depend on different sources to comprehend and reduce the burden of relying on single source (Mayer, 2014). In effect, this allows the brain to process more information in the working memory and retain them whenever required from the long-term memory (Sweller, 2005). YouTube videos with captions and their adjustable settings (i.e., font size and colour) were proposed in this work to create and enhance a multimedia (both visual and verbal) learning environment for the development of ESL writing accuracy.

#### 2.1.2 Signalling principle

Mayer (2009) stated that adding signals or cues to highlight learning materials can result in a better learning. That said, learners need to know where to direct their attention particularly if there are many information presented to them on-screen. As such instructors are recommended to include signals to direct learners' attention and make learning materials more salient. In this regard, while using YouTube captioned videos, teachers can highlight and draw learners' attention to important information (i.e., their errors/gaps) in hand using the adjustable settings of font and colour which can be adjusted for that matter. These features will not only facilitate comprehension of the target language input but also draw learners' attention to their errors/gaps and support retention of language points in hand.

#### 2.1.3 Temporal contiguity principle

It involves presenting information (as in text, audio, pictures, and video/animation) synchronously rather than in sequence; this results in a greater depth of learning (Mayer and Fiorella, 2014, as cited in Kanellopoulou et al., 2019). In this regard, YouTube can provide learners with captions of a given spoken learning material in real time.

#### 2.1.4 Noticing the gap hypothesis

Schmidt (1990, 2001) stated that target language input does not become intake for language learning unless it is noticed (i.e., consciously registered), and that in order to overcome errors, learners must make conscious comparisons between their own output and target language input. In this regard, the proposed affordances of YouTube video captions and adjustable settings of font size and colour can be helpful if utilized for their multimedia effects, i.e., to draw the learners' attention to the target language input (i.e., for conscious comparisons) and to help them clearly notice their errors/gaps and make conscious comparisons between their own output and target language input.

## 3 Materials and methods

### 3.1 Context of study

The main aim of this work was to examine the role and impact of ICT tools such as YouTube with respect to its affordances of captions and their adjustable settings like font size and colour (i.e., enhanced captions of videos) on the development of ESL learners' writing accuracy over time, given that they are exposed to ICT multimedia learning tools like YouTube during their learning process. The first objective of this study was to determine the impact of the frequent use of the YouTube affordances (i.e., enhanced captions of YouTube videos) on the development of ESL learners' writing accuracy. In this respect, different outcomes were set by the questionnaire (Tables 1 and 4). The second objective was to determine if the writing accuracy of English learners improved after the implementation of the proposed YouTube affordances for the improvement of ESL writing accuracy. In this regard, different outcomes were measured by the repeated measures test (Table 2). The third objective was to determine the correlation between the learners' L2 writing accuracy improvement, if any, and the frequent use of the proposed YouTube affordances for the development of ESL writing accuracy. Different outcomes were measured by the correlation test (Table 3).

This work is a longitudinal study which explored written language features of a single group of learners using repeated measures design, where the same individuals were measured on a number (as few as two) of occasions as suggested by Zmyslinski-Seelig (2017). Technically, the design was a One-Group Pretest-Posttest Design (O_1_ X_1_ O_2_). In this design, the effect of a treatment (X_1_) was determined by calculating the difference between the first assessment of the dependent variable (i.e., the pretest or O_1_) and the second assessment of the dependent variable (i.e., the posttest or O_2_). This design was beneficial and suitable to conduct this study as there was only one group of participants (i.e., the treatment group) available to the researcher and also to create a control group (who absolutely had strictly no access to YouTube or training) was unethical because all members of this group showed interest in this project and wanted to improve their L2 writing accuracy (Cranmer, 2017). The experiment^3^ spanned over five months (December 2018 to April 2019) and was based at the Iraqi school in Delhi where a group of students (*n.* 14) whose age was 15 years participated in this study. They were five boys and nine girls, i.e., 35% and 65%, respectively. These participants showed interest in this project and thus were randomly selected and included (and no one was excluded) in this study. The language proficiency level of this group of learners was around intermediate as indicated by their formative assessment scores of the English subject. Arabic language was the medium of instruction at the school except for English classes which were mostly taught in English. English was the second language for this group of learners as long as they lived in Delhi where English “for most of the population has only ever been a second language” (Robinson, 2019). Initially, the general framework of this experiment was explained to the participants. Also, participants were constantly requested and reminded to solely depend on the proposed learning method in this study (especially while at home) as far as English writing improvement was concerned. In other words, participants were requested to adhere to the prescribed procedures given to them to ensure that other learning sources or what is technically known as confounding variables were controlled and were not intervening in the development of the target variable, i.e., learners' L2 writing accuracy. Thus, the author could make a valid judgment about the potential role and likely impact of YouTube on the development of learners' writing accuracy for this sample group. The researcher met the participants for forty-five minutes three times weekly (holidays were compensated either the day before or after the holiday) over five months. It should be mentioned that this period was a full-semester of the academic year at this school. This period was deemed to be enough for these learners to try out the proposed method and experience its potential advantages. Ortega and Iberri-Shea (2005) suggested that “most recent longitudinal SLA studies span anywhere between three or four months and up to six years”. In every meeting, learners were introduced to a new topic using some YouTube video to learn from. The total number of videos introduced to learners in this study was 60 videos. The themes of the stories used in these videos covered a multitude of topics related to various types of sports, health issues, cultural differences, foods and drinks, languages, societal problems and solutions, future plans, technology and innovation among many other topics of learners' particular interest. YouTube was at the core of every English class to learn from its videos content in ‘a watch-take notes-discuss’ learning modality. The BBC Six-Minute English YouTube channel was proposed by the author as it was only a six-minute show designed for intermediate learners of English, and it matched the learners' proficiency level. Basically, the writing tasks and activities given to participants were structured around the language content and topics of these YouTube videos. The English used in these videos was General English presented in an informal and conversational style to help learners practice authentic English in their daily lives, i.e., writing something in English. General English helps learners “achieve a high standard of everyday English communication skills; it covers the four main skills of reading, writing, listening and speaking” (University of Western Australia, n.d.). The presenters in this YouTube channel teach language that learners can use to discuss and write about something like a short story, an essay or a letter.

### 3.2 Procedures

This section describes how the proposed affordances of YouTube (i.e., enhanced captions) were employed by the learners and researcher across the three writing stages (i.e., pre-writing, revision and proofreading stages) while using YouTube videos for ESL writing tasks both at home and inside English writing classes over five months.

#### 3.2.1 Homework

Three different videos about different topics were suggested at the end of every English writing class, and participants had to choose one of them to be the subject matter for the next class. Regarding the pre-writing stage, initially participants were requested to make use of the enhanced captions of videos to comprehend the chosen topic of a given video on their own at home. Also, for discussing the topic in the writing task given to learners, they were encouraged to try to make use of the new target language input (used by the presenters in a given video to introduce a new topic) in terms of both content (i.e., lexical items) and forms (i.e., grammatical/morphological structures and mechanics of writing). Writing tasks, which were structured around these videos content, involved various writing activities (i.e., summaries, letters, and short essays) depending on the nature of the topic of a given video. Besides, they were encouraged to write their notes, comments and inquiries about the topic to be discussed openly with the whole class. As for the revision and proofreading stages (i.e., after they have finished a given writing activity), participants were also encouraged to use the enhanced captions technique to check on (i.e., by way of conscious comparisons between their own output and target language input) the right meaning of some sentence in a given context or to check problematic/unfamiliar grammatical forms/vocabulary, the accuracy of word choice, morphological/grammatical structures, abbreviations, spelling, punctuation or capitalization they came across while they were learning using some YouTube video. Participants had to do this on their own at home for every writing task.

#### 3.2.2 Class work

The assigned video with captions turned on was viewed again by all learners and the instructor^4^ at the beginning of the writing class. Afterwards, learners were requested to read out their writings and discuss the overall idea of the new topic. Regarding fidelity checks on the assigned homework, the instructor would ask few questions related to the assigned video content and would invite learners to answer and comment on them. Answering these questions would require that the participants have spent a good deal of time at home using the proposed method of learning in this study. The instructor would use the captions and adjustable settings of font size and colour for their multimedia and signalling effects, i.e., to increase the learners' attention especially to the (new) target language input and their errors/gaps, process more information in the working memory, increase their comprehension of the spoken language input and retain these information whenever required from the long-term memory. The captions would be projected on the board for referring to and highlighting interesting and significant language points/inquiries such as those directly raised by the learners themselves, or related to learners' errors in their English writing and noticed by the learners and/or the instructor. Also, the instructor would point out some language points (i.e., using the enhanced captions for that matter) whenever found noteworthy, relevant and enriching for the learners' writing experience. In other words, with regard to fidelity checks in terms of class work, the instructor would make sure to adhere to the proposed method and engage participants with the class work using the proposed method. Again, participants were reminded to use the captioning to support them whenever they needed for the same reasons mentioned above (i.e., Homework section). Apparently, the classwork was all about revising and proofreading learners' writings (which were done at home) depending on the target language input of the suggested YouTube videos content while using the enhanced captions as a technique for learning more about English writing and improving its accuracy.

### 3.3 Data collection

First, International English Language Testing System (IELTS)-based communicative writing tasks^5^ (i.e., which involved writing a letter and short essay about a topic of general interests) were given to participants to set the baseline before the experiment, and the same examination procedure was repeated (while using different topics for the same type of the writing tasks) after five months of the integration of YouTube to set the end line of this study. More specifically, learners were examined on two communicative writing tasks each time (i.e., both before and after the intervention) rather than a single one (i.e., more representative of the learners' overall communicative writing), and the total of the tasks scores for each learner was averaged and taken as a single line of reference each time (for each of the baseline and end line tests) for final evaluation. The aim of these two benchmarks was to determine whether or not learners' writing accuracy developed after the implementation of the proposed YouTube affordances in the writing classes; technically, this could be done by assessing/analysing the variance (ANOVA) in the participants' scores before as opposed to after the intervention. These tests provided data to answer the second research question in this study (Table 2) (i.e., the second objective in this study). For information on the five-month intervention period and proposed techniques to operationalize learners' writing accuracy in this study, see the procedures section above 3.2. It should be noted that the author adopted this IELTS test for the assessment of learners' L2 writing after discussing the assessment methods and materials (and the learners' test scores later on) with four linguists who are quite familiar with the IELTS test (see the inter-rater reliability below, Data analysis 3.4.).

Second, an online self-report questionnaire (Tables 1 and 4), which combined both quantitative and qualitative items, was adapted from Alobaid (2020) and partially developed by the author according to the specific aims and context of this study following Dörnyei and Csizér (2012) for designing and analysing surveys in second language acquisition research. The questionnaire was designed with a deliberate focus on learners' personal experience in terms of the benefits gained from their actual use and potential advantages of YouTube captioned videos proposed for ESL writing accuracy development. It was used to determine the role and impact of ICT tools like YouTube on the development of ESL writing accuracy over five months. This survey was administered online at the end of the five months of this focused study. Learners were asked to consider the suggested BBC six-minute English YouTube channel while answering the questionnaire questions. Participants' responses to the questionnaire items were used to answer the first research question (i.e., the first objective in this study). This questionnaire (9 items) combined both closed-ended and Likert questions. The questionnaire items Q1, Q2, Q3, Q5, Q6, Q7, Q8, Q9 (see the frequency distribution analysis, Table 1) provided data for the quantitative analysis part of this study (i.e., frequency distribution and correlation tests). Question 4 (see the content analysis, Table 4) provided data for the qualitative analysis part.

### 3.4 Data analysis

This author used six quantifiable measurements as indicators of writing accuracy development (i.e., these were employed as the dependent variables for the correlational analysis in this study), namely *number of errors per T-unit*^6^ (NEPTU), where the number of errors were divided by the total number of T-units in the text as suggested by Kuiken and Vedder (2008)*; number of errors per 100 words* (NEP100Ws), where the number of errors were divided by the total number of the produced words then divided by 100 as suggested by Bygate (2001); *target-like use of vocabulary*^7^ (TLUV), where the number of lexical errors were divided by the total number of words in the text, excluding dysfluencies as suggested by Skehan and Foster (1997)*; percentage of target-like verbal morphology* (PTLVM), where the number of correct finite verb phrases were divided by the total number of verb phrases then multiplied by 100 as suggested by Wigglesworth (1997)*; percentage of target-like use of articles* (PTLUA), where the number of correctly used articles were divided by the obligatory occasions for articles then multiplied by 100 as suggested by Crookes (1989); *ratio of error-free clauses* (REFCs)*,* where the number of error free clauses were divided by total number of independent clauses, subclausal units and subordinate clauses then multiplied by 100 as suggested by Foster and Skehan (1996).

As for the writing tasks evaluation and the error analysis done in this regard, all participants' responses (*n.* 14) were objectively analysed in terms of writing accuracy using the taxonomy of errors suggested by Dulay (1982) and James (1998) as cited in Ellis and Barkhuizen (2005). This taxonomy involved grammatical and morphological aspects (i.e., omission, addition, misinformation, misordering and blends) and aspects related to mechanics of writing like misspelling, capitalization and punctuation. Furthermore, learners' errors were coded following (Oshima and Hogue, 1997; Ferdouse, 2013). Also, it should be mentioned that the kind of errors used in this work were both data-driven and theoretically motivated. Each writing task was rated by four specialists in the field and the inter-rater reliability analysis for the total writing tasks (*n.* 56) showed Cronbach's alpha α = 0.81. As a general rule, α of 0.8 or greater indicates a very good level of reliability (Hulin et al., 2001). Data elicited from the writing tasks of the repeated-measures design and the questionnaire outcomes for all learners' responses (*n.* 14) were tabulated into Microsoft Excel sheets for processing and coding and subsequently exported to SPSS version 21.0 for running the concerned statistical analyses.

Datasets were analysed quantitatively and qualitatively. The quantitative part included Frequency distributions, Wilcoxon matched-pairs ranked test and Spearman's correlation. Frequency distributions analysis was employed to analyze the learners' responses to the questionnaire items in order to determine the role and impact of the frequent use of the enhanced captions of YouTube videos on the learners' ESL writing accuracy development. Frequency distributions were run on learners' responses to the questionnaire for the dichotomous (Yes/No) and rating scale (1–5 negative to positive ratings) questions as the initial stage of the quantitative analyses. To account for any potential variation between the baseline and end line test scores of learners' writing performance, Wilcoxon matched-pairs ranked test was employed. This test is suitable when the same participants are assessed under two different conditions, i.e., repeated measure design as the case in this study (Scheff, 2016). It is the nonparametric equivalent of the parametric paired *t*-test; it was used as the chief assumption of the parametric matched *t* test of normally distributed data was not met due to the small sample size of this group of learners. Spearman's ranked order correlation was used to examine the relationship between the frequent use of the enhanced captions of YouTube videos for the development of the ESL learners' writing accuracy (i.e., this represented the independent variables for this correlational analysis whose data were elicited from learners' responses to the questionnaire items provided in Table 1) and the learners' writing accuracy development, if any, after the implementation of the proposed YouTube affordances for the improvement of ESL writing accuracy (i.e., this represented the dependent variables for this correlational analysis whose data were the learners' scores of the post-test writing tasks provided in Table 2). In other words, this analysis was run to identify the correlation between the first research question and the second research question; the findings of this analysis provided the answer to the third research question in this study (i.e., third objective). Also, it should be mentioned that Spearman's correlation is a non-parametric correlation coefficient test which was used in this study as the normality and randomness assumptions of data were not met (Bastick and Matalon, 2007) and because when one or both of the variables are ordered categorical rather than scaled variables as the case in this study, the test may be based on Spearman's rank correlation coefficient (McPherson, 2013).

Finally, the qualitative part was based on Content analysis which was run using one closed-ended question (Q4, Table 4). This question was a follow-up to the questionnaire item (Q3, Table 1) about the extent to which learners thought YouTube multimedia affordances (i.e., enhanced captions of videos) made the process of learning about and improving their English writing accuracy easy. Participants were required to tick the given choices according to their actual experience. The provided choices, which were designed by the author for the purpose of this study and were in line with the main research questions in this work, are widely discussed in the literature of L2 writing development (see Discussion section 5.1.).

## 4 Results

### 4.1 Quantitative results

#### 4.1.1 Frequency distributions analysis

This analysis was used to assess the questionnaire findings in order to determine the role and impact of the frequent use of the enhanced captions of YouTube videos on the ESL learners' writing accuracy development over five months (i.e., the first objective in this study). Frequency distributions findings (Table 1) suggested that the frequent use of ICT multimedia tools like YouTube with respect to its affordances of captions and their adjustable settings like font size and colour (i.e., enhanced captions of videos) played a positive role that impacted the improvement of learners' English writing accuracy over five months.

The following are the frequency distributions results of this study which can be found in more details in (Table 1).

**Table 1:** Frequency distributions of participants' responses to the questionnaire items (*n* = 14).

Items	Frequency	Percent
Q1. Did you use the captioning and the related settings on YouTube, i.e., font size & colour inside and outside the writing classes to help you learn about and improve English writing?		
Yes	12	86%
No	2	14%
Q2. How often did you use the captioning and the adjustable related settings on YouTube, i.e., font size & colour inside and outside the writing classes to help you learn about and improve English writing?		
Never	0	0
Rarely	0	0
Sometimes	2	14%
Very often	9	65%
Always	3	21%
Q3. Rate the extent to which the frequent use of the captioning and the adjustable related settings on YouTube, i.e., font size & colour inside and outside the writing classes made learning about and improving English writing easy.		
Not easy	0	0
Fairly easy	0	0
Easy	1	7%
Very easy	6	43%
Extremely easy	7	50%
Q5. Rate the extent to which the frequent use of the captioning and the adjustable related settings on YouTube, i.e., font size & colour inside and outside the writing classes were helpful or not helpful for learning about and improving English writing.		
Not helpful	0	0
Fairly helpful	2	14%
Helpful	1	7%
Very helpful	8	58%
Extremely helpful	3	21%
Q6. Rate the extent to which the frequent use of the enhanced captions of YouTube videos benefited you to learn about and improve your English writing accuracy in terms of correct vocabulary and language expressions in context.		
No improvement	0	0
Little improvement	0	0
Some improvement	3	21%
Much improvement	7	50%
Very much improvement	4	29%
Q7. Rate the extent to which the frequent use of the enhanced captions of YouTube videos benefited you to learn about and improve your English writing accuracy in terms of correct grammatical/morphological forms and structures.		
No improvement	0	0
Little improvement	0	0
Some improvement	3	21%
Much improvement	8	58%
Very much improvement	3	21%
Q8. Rate the extent to which the frequent use of the enhanced captions of YouTube videos benefited you to learn about and improve your English writing accuracy in terms of correct spelling, capitalization and punctuations.		
No improvement	0	0
Little improvement	0	0
Some improvement	2	14%
Much improvement	8	58%
Very much improvement	4	28%
Q9. Do you usually practice the stuff you have learnt with the help of enhanced captions of YouTube videos when you write in English?		
Yes	11	79%
No	3	21%

Question (Q.1) was intended to know if learners used the captioning and the adjustable settings on YouTube, i.e., font size & colour inside and outside the class to help them learn about and improve English writing; nearly all participants (86%) answered with “Yes”. Learners were asked question (Q2) as a follow-up to question (Q1) to determine how often they used the captioning and the adjustable settings on YouTube, i.e., font size & colour inside and outside the writing classes to help them learn about and improve English writing; more than half of them (65%) answered “very often” and 14 % & 21 % of them answered “sometimes” and “always”, respectively. Question (Q3) revealed that the use of the captioning and the adjustable settings on YouTube, i.e., font size & colour made learning about and improving English writing “extremely easy” for 50% of learners, “very easy” and “easy” for 43% and 7% of them, respectively. Question (Q5) was about the degree of helpfulness of the use of the captioning and the adjustable settings on YouTube, i.e., font size & colour to help learners learn about and improve English writing; more than half of the participants (58%) found them “very helpful”, 21%, 7 %, 14 % of participants found them “fairly helpful”, “helpful” and “extremely helpful”, respectively. Participants' responses to question (Q6), which was related to their writing accuracy improvement in terms of correct vocabulary and language expressions in context due to the frequent use of the enhanced captions of YouTube videos used for ESL writing accuracy development, showed that half of them (50%) reported “much improvement”, 21% “some improvement” and 29% “very much improvement”. Responses to question (Q7), which was related to participants' writing improvement in terms of correct grammatical/morphological forms and structures due to the use of the enhanced captions of YouTube videos used for ESL writing accuracy development, revealed that more than half of the participants (58%) reported “much improvement”, 21% “some improvement” and 21% “very much improvement”. Responses to question (Q8), which was related to learners' writing development in terms of correct spelling, capitalization and punctuation due to the use of the enhanced captions of YouTube videos used for ESL writing accuracy development, revealed that more than half of the participants (58%) reported “much improvement”, 14% “some improvement” and 28% of them “very much improvement”. Regarding question (Q9), which was a follow-up to the previous three questions, about the actual use and practice of the stuff learners have learnt with the help of enhanced captions of YouTube videos when they write in English; the vast majority of participants' responses (79%) were in the affirmative “Yes”.

#### 4.1.2 Wilcoxon Matched-Pairs Ranked Test

This test was used to account for any potential variation between the baseline and end line test scores of learners' writing performance. The aim of this test was to determine whether or not learners' writing accuracy developed after the implementation of the proposed YouTube affordances in the writing classes (i.e., the second objective in this study). In this study, the critical *Z* value for a 95% confidence interval was used for hypothesis testing. For a 2-tailed test (which was the case in this study) if *Z* < -1.96 or *Z* > +1.96, the null hypothesis (see the literature review section 2.) set in this work could be rejected.

**Figure 1:**
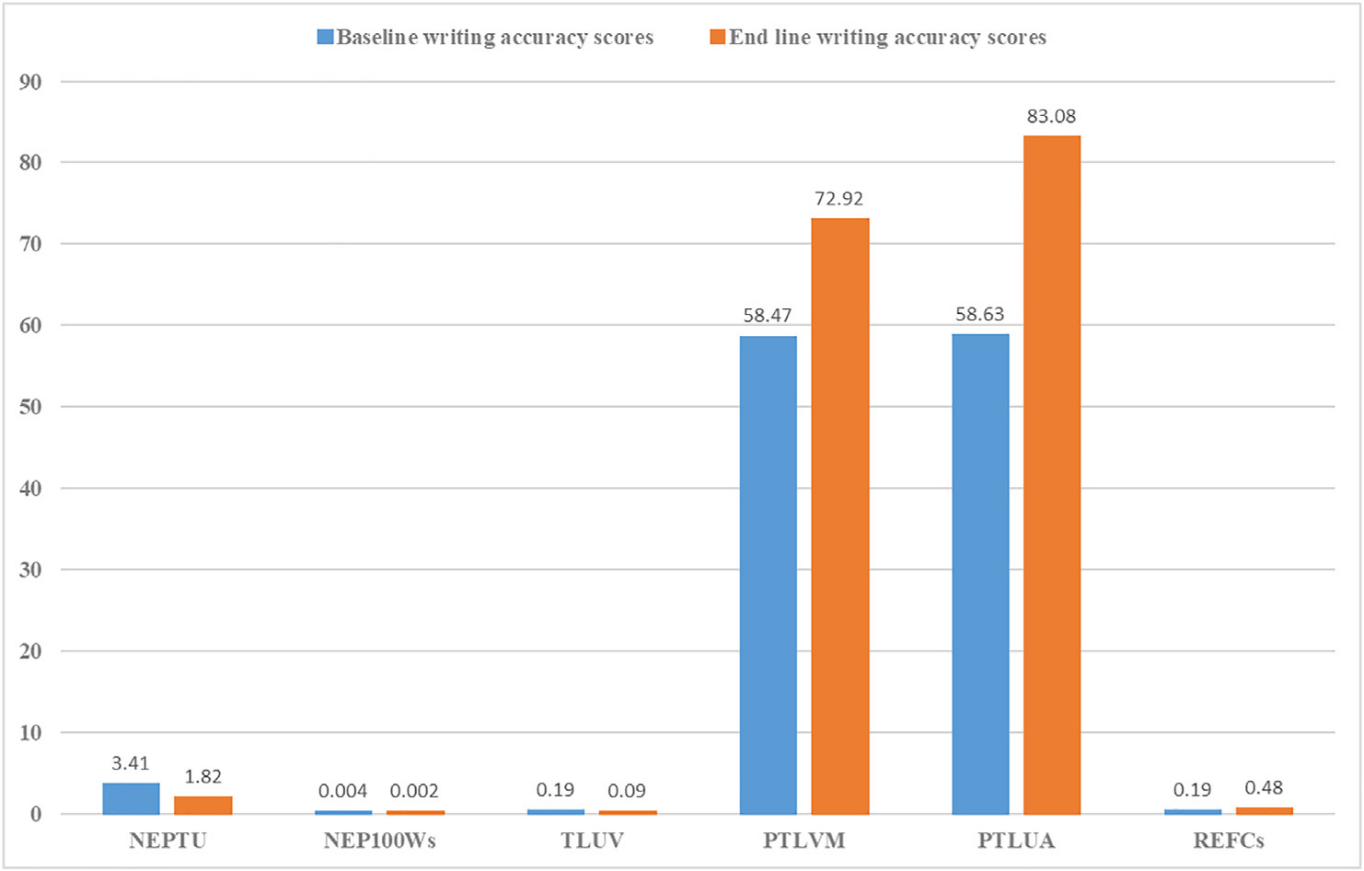
The baseline and end line test scores of learners' writing accuracy performance showing the likely impact of YouTube enhanced-captioned videos on ESL learners' writing accuracy development.

The results (Table 2) revealed statistically significant differences between the medians of this group of learners before and after the implementation of the proposed YouTube affordances for the improvement of ESL writing accuracy as shown by all of the *p* values (*p* < 0.001) of this test. Also, given that the *Z* scores in this test were below -1.96, this suggested that the null hypothesis could be rejected in favour of the alternative hypothesis. Moreover, the effect size of this test was found to exceed Cohen's (1988) convention for a medium effect (*d* = .50). Considering these significant results of this test, it could be concluded that some improvement in the accuracy of English writing was made by this group of learners after five months of the implementation of the proposed YouTube affordances for the improvement of ESL learners' writing accuracy (see Figure 1).

**Table 2:** Summary table of Wilcoxon matched-pairs ranked test results between Baseline and End line of learners' writing performance.

Writing Accuracy Metrics	Baseline test scores: mean (sd)	End line test scores: mean (sd)	(End line-score) − (Baseline-score)				Wilcoxon Signed Rank		Effect Size
			Neg. Rank	Posit. Rank	Ties	Total	Z	P-Value	
No. of errors per T-Unit (NEPTU)	3.41 (.99)	1.82 (1.06)	14	0	0	14	-3.296	0.001	-0.62
No. of errors per 100 words (NEP100Ws)	.0042 (.002)	.0020 (.001)	14	0	0	14	-3.315	0.001	-0.63
Target-like use of vocabulary (TLUV)	0.19 (.12)	0.09 (.06)	14	0	0	14	-3.3	0.001	-0.62
Percentage of target-like verbal morphology (PTLVM)	57.87 (10.78)	72.92 (6.98)	0	14	0	14	-3.296	0.001	-0.62
Percentage of target-like use of articles (PTLUA)	58.63 (28.26)	83.08 (13.99)	1	13	0	14	-3.233	0.001	-0.61
Ratio of error-free clauses (REFCs)	.19 (.19)	.48 (.24)	0	14	0	14	-3.296	0.001	-0.62

The following are the Wilcoxon test results in more details.

- **Number of errors per T-unit (NEPTU)**

A Wilcoxon Matched-Pairs Ranked Test indicated that the post-test scores were statistically significantly higher than pre-test scores in terms of the *number of errors per T-unit Z* = -3.296, *p* < 0.001, *d* = -0.62.

- **Number of errors per 100 words (NEP100Ws)**

A Wilcoxon Matched-Pairs Ranked Test indicated that the post-test scores were statistically significantly higher than pre-test scores in terms of the *number of errors per 100 words Z* = -3.315, *p* < 0.001, *d* = -0.63.

- **Target-like use of vocabulary (TLUV)**

A Wilcoxon Matched-Pairs Ranked Test indicated that the post-test scores were statistically significantly higher than pre-test scores in terms of the *target-like use of vocabulary Z* = -3.3, *p* < 0.001, *d* = -0.62.

- **Percentage of target-like verbal morphology (PTLVM)**

A Wilcoxon Matched-Pairs Ranked Test indicated that the post-test scores were statistically significantly higher than pre-test scores in terms of the *percentage of target-like verbal morphology Z* = -3.296, *p* < 0.001, *d* = -0.62.

- **Percentage of target-like use of articles (PTLUA)**

A Wilcoxon Matched-Pairs Ranked indicated that the post-test scores were statistically significantly higher than pre-test scores in terms of the *percentage of target-like use of articles Z* = -3.233, *p* < 0.001, *d* = -0.61.

- **Ratio of error-free clauses (REFCs)**

A Wilcoxon Matched-Pairs Ranked Test indicated that the post-test scores were statistically significantly higher than pre-test scores in terms of the *ratio of error-free clauses* Z = -3.296, *p* < 0.001, *d* = -0.62.

Given that some improvement in the accuracy of writing was made by this group of learners after five months of the implementation of the proposed YouTube affordances for the improvement of ESL learners' writing accuracy (as indicated above by all the metrics used in this study for measuring the learners' writing accuracy progress), this encouraged running the following correlational analysis to check whether this improvement was associated with the positive impact of the frequent use of the enhanced captions of YouTube videos on the ESL learners' writing accuracy development seen in (Table 1).

#### 4.1.3 Spearman's rank correlation coefficient test (the critical value approach)

This statistical test was used to determine the correlation between the learners' L2 writing accuracy improvement see in (Table 2) and the positive impact of the frequent use of the enhanced captions of YouTube videos on the ESL learners' writing accuracy development seen in (Table 1). More specifically, this test explored the strength and direction of the relationship between the concerned variables in this study; the independent variable being the frequent use of the enhanced captions of YouTube videos as a technique for the development of the ESL learners' writing accuracy, and the dependent variable being the learners' writing accuracy development, if any, post the implementation of the proposed YouTube affordances as a technique for the improvement of the learners' writing accuracy (i.e., the third research question in this study). As for the strength of relationship between these variables, the critical value for Spearman |r| was set at 0.05 = 0.464. This was the significance level for a two-tailed test which can be found in the critical values table of the Spearman's ranked correlation coefficient (*r*_*s*_) (Zar, 1984, Table B.19). The degree of freedom df = N-2, df = 14–2 = 12. Correlation coefficient values below the 0. 464 were considered insignificant, while those above the 0. 464 were significant. The higher the *r* value or closer to |+1/-1|, the stronger the correlation is between two variables. Consequently, the better the learners' writing accuracy is.

Regarding the direction (i.e., negative = -ve/positive = +ve) of relationship between these variables, the first three writing accuracy metrics (dependent variables) in the correlation table (Table 3), namely *number of errors per T-unit*, *number of errors per 100 words* and *the target-like use of vocabulary* should have been negatively correlated with the independent variables since these dependent variables dealt with errors made by the learners; the lower the number of errors means the higher writing accuracy is. As far as these three metrics were concerned, the correlation was, as predicted before the study, a negative correlation, i.e., a relationship in which an increase in one variable (in this case, the frequent use of the enhanced captions of YouTube videos for the development of ESL learners' writing accuracy) was associated with a decrease in the other variable (in this case the number of writing errors made by learners). However, the last three writing accuracy metrics (dependent variables) in the correlation table (Table 3) should have been positively correlated with the independent variables as these dependent variables dealt with *percentages of target-like use of verbal morphology/articles* and *ratio of error-free clauses*. As far as these three metrics were concerned, the correlation was, as predicted before the study, a positive correlation, i.e., a relationship in which one variable increases (in this case, the frequent use of the enhanced captions of YouTube videos for the development of ESL learners' writing accuracy) as the other variable increases (in this case the percentages of the target-like use of learners' language patterns).

**Figure 2:**
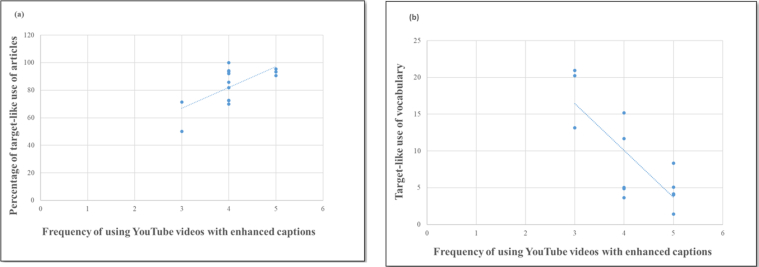
Two samples of moderately positive linear correlation (a) and strongly negative linear correlation (b).

This test was found to be statistically significant. The findings (Table 3) indicated linear relationships (i.e., the direction aspect of correlation) between the concerned variables in this study. Also, these findings demonstrated a range of statistically significant correlations (moderate & strong correlations, i.e., the strength aspect of correlation) between the development of learners' L2 writing accuracy and the frequent use of the YouTube affordances of enhanced captions as a technique for the development of ESL learners' writing accuracy (see Figure 2 a & b).

Below are the results of only the statistically significant correlation coefficients (*r*_*s*_) and (*p*) values of this test presented row-wise as ordered in (Table 3).

- Results (Row 1) of participants' responses to questions Q2, Q5, Q6, Q8 demonstrated significant negative correlations with their writing accuracy development in terms of the *number of errors per T-unit* after the learners started using the enhanced captions of YouTube videos frequently as a technique for the improvement of their writing accuracy (*r*_*s*_ = -.59, *p* = .026); (*r*_*s*_ = -.57, *p* = .034); (*r*_*s*_ = -.64, *p* = .013); (*r*_*s*_ = -.64, *p* = .014), respectively.
- Results (Row 2) of participants' responses to questions Q1, Q2, Q5, Q6, Q7, Q8, Q9 demonstrated significant negative correlations with their writing accuracy development in terms of the *number of errors per 100 words* after the learners started using the enhanced captions of YouTube videos frequently as a technique for the improvement of their writing accuracy (*r*_*s*_ = -.60, *p* = .023); (*r*_*s*_ = -.66, *p* = .010); (*r*_*s*_ = -.62, *p* = .018); (*r*_*s*_ = -.71, *p* = .004); (*r*_*s*_ = -.53, *p* =. 049); (*r*_*s*_ = -.69, *p* = .007); (*r*_*s*_ = -.59, *p* = .027), respectively.
- Results (Row 3) of participants' responses to questions Q2, Q3, Q6, Q7, Q8, Q9 demonstrated significant negative correlations with their writing accuracy development in terms of the *target-like use of vocabulary* after the learners started using the enhanced captions of YouTube videos frequently as a technique for the improvement of their writing accuracy (*r*_*s*_ = -.58, *p* = .031); (*r*_*s*_ = -.56, *p* = .039); (*r*_*s*_ = -.68, *p* = .007); (*r*_*s*_ = - .54, *p* = .046); (*r*_*s*_ = -.62, *p* = .017); (*r*_*s*_ = -.71, *p* = .004), respectively.
- Results (Row 4) of participants' responses to question Q1 demonstrated significant positive correlation with their writing accuracy development in terms of the percentage of the *target-like verbal morphology* after the learners started using the enhanced captions of YouTube videos frequently as a technique for the improvement of their writing accuracy (*r*_*s*_ = .56, *p* = .039).
- Results (Row 5) of participants' responses to questions Q1, Q3, Q5, Q6, Q7, Q8, Q9 demonstrated significant positive correlations with their writing accuracy development in terms of the *percentage of the target-like use of articles* after the learners started using the enhanced captions of YouTube videos frequently as a technique for the improvement of their writing accuracy (*r*_*s*_ = .58, *p* = .031); (*r*_*s*_ = .56, *p* = .039); (*r*_*s*_ = .62, *p* = .017); (*r*_*s*_ = .74, *p* = .002); (*r*_*s*_ = .62, *p* = .017); (*r*_*s*_ = .70, *p* = .006); (*r*_*s*_ = .58, *p* = .029), respectively.
- Results (Row 6) of participants' responses to questions Q1, Q2, Q3, Q5, Q6, Q7, Q8, Q9 demonstrated significant positive correlations with their writing accuracy development in terms of the *ratio of error-free clauses* after the learners started using the enhanced captions of YouTube videos frequently as a technique for the improvement of their writing accuracy (*r*_*s*_ = .67, *p* .009); (*r*_*s*_ = .61, *p* .021); (*r*_*s*_ = .61, *p* .021); (*r*_*s*_ = .65, *p* .011); (*r*_*s*_ = .79, *p* .001); (*r*_*s*_ = .65, *p* .012); (*r*_*s*_ = .72, *p* .004); (*r*_*s*_ = .71, *p* .004), respectively.

**Table 3:** Nonparametric Correlations (Spearman's rho) between YouTube multimedia affordances and learners' perspectives on its use inside and outside writing classes and learners' writing accuracy development.

			Q1	Q2	Q3	Q5	Q6	Q7	Q8	Q9
Writing Accuracy Metrics	No. of Errors per T-Unit	Correlation Coefficient (rs)	-0.490	-.590∗	-0.405	-.569∗	-.643∗	-0.514	-.637∗	-0.497
		Sig. (p)	0.075	0.026	0.151	0.034	0.013	0.060	0.014	0.071
	No. of errors per 100 words	Correlation Coefficient (rs)	-.600∗	-.662∗∗	-0.512	-.618∗	-.712∗∗	-.534∗	-.685∗∗	-.589∗
		Sig. (p)	0.023	0.010	0.061	0.018	0.004	0.049	0.007	0.027
	Target-like use of vocabulary	Correlation Coefficient (rs)	-0.421	-.575∗	-.557∗	-0.527	-.683∗∗	-.541∗	-.623∗	-.713∗∗
		Sig. (p)	0.134	0.031	0.039	0.053	0.007	0.046	0.017	0.004
	Percentage of target-like verbal morphology	Correlation Coefficient (rs)	.555∗	0.398	0.354	0.458	0.516	0.406	0.524	0.410
		Sig. (p)	0.039	0.159	0.214	0.099	0.059	0.150	0.055	0.145
	Percentage of target-like use of articles	Correlation Coefficient (rs)	.575∗	0.447	.557∗	.622∗	.743∗∗	.623∗	.697∗∗	.583∗
		Sig. (p)	0.031	0.109	0.039	0.017	0.002	0.017	0.006	0.029
	Ratio of error-free clauses	Correlation Coefficient (rs)	.666∗∗	.607∗	.608∗	.654∗	.788∗∗	.650∗	.717∗∗	.713∗∗
		Sig. (p)	0.009	0.021	0.021	0.011	0.001	0.012	0.004	0.004

∗. Correlation is significant at the 0.05 level (2-tailed).

∗∗. Correlation is significant at the 0.01 level (2-tailed).

### 4.2 Qualitative results

#### 4.2.1 Content analysis

The findings of this analysis supported and added value to the quantitative findings in this study and brought more evidence about the potential positive impact of the frequent use of the enhanced captions of YouTube videos on the development of learners' L2 writing accuracy. This was demonstrated through the participants' personal experience with the proposed method in terms of the benefits gained from the actual use and potential advantages of the enhanced captions of YouTube videos which were used as a technique for the development of writing accuracy of ESL learners (Table 4). This table includes participants' responses in the affirmative (given in numbers and percentages) to the closed-question (Q.4) in the questionnaire (see Table 4).

**Table 4:** Represents the extent to which learners thought YouTube multimedia affordances (i.e., the enhanced captions of videos) made learning about and improving their L2 writing accuracy easy.

Q4. How do you think the enhanced captions of YouTube videos made learning and improving English writing easy for you?	(*n*) % responses	Implications
a- The enhanced captions can make learning writing easier because it can optimize comprehension of YouTube videos content/topics, especially in the pre-writing stage.	(14) 100%	This shows that enhanced captioning can be helpful to make videos content and language materials more comprehensible for learners before they start writing.
b- The enhanced captions attract and focus the learners' attention on their writing errors or gaps; this helps me notice, correct and learn from my mistakes (especially my vocabulary, grammar and spelling mistakes) and ultimately improve my overall language use of English writing, i.e., through checking my writing with the language used in some video content (given that the language used in some video is taken as a model, especially the language of native speakers), especially in the revision and proofreading stage of writing.	(13) 92%	This shows the significant role of enhanced captions for learners to notice their errors and learn from them when they revise and proofread their writing.
c- The enhanced captions attract and focus the learners' attention on new language points related to the target language use of grammar, vocabulary, spelling and punctuations; this can facilitate the retention of the newly learnt information and the overall process of learning and improvement of L2 writing.	(12) 85%	This shows that enhanced captions can be used to help draw learners' attention and improve retention of newly learnt language and ultimately contributing to their overall L2 writing accuracy improvement.
d- The enhanced captions create and enhance the multimedia learning environment for different learning styles; they are especially supportive for the visual and/or auditory learner.	(13) 92%	This demonstrates the assistive role of enhanced captions to cater for different learning styles.

*Note.* Adapted from *Smart multimedia learning of ICT: role and impact on language learners' writing fluency— YouTube online English learning resources as an example*, by Azzam Alobaid. Retrieved from https://doi.org/10.1186/s40561-020-00134-7 Copyright 2020, Springer Nature.

## 5 Discussion

There has been a huge body of research with emphasis on the affordances of ICT multimedia tools like YouTube with reference to its role and impact on the development of ESL/EFL receptive and productive skills. This work extended the research in this area by exploring the potential role and impact of multimedia affordances of YouTube as an ICT multimedia learning/teaching tool with respect to its affordances of captions and their adjustable settings of font size and colour (i.e., enhanced captions of videos) to create and enhance a multimedia learning environment with a special focus on the writing accuracy development of ESL learners.

With regard to the first research question in this study, the quantitative findings (Table 1) about learners' personal experiences in terms of the benefits gained from the actual use and potential advantages of the enhanced captions of YouTube videos used for ESL writing accuracy development clearly revealed that the frequent use of ICT multimedia tools like YouTube affordances of videos with captions and their adjustable settings (i.e., enhanced captions) played a positive role that impacted the improvement of learners' English writing accuracy over five months. Specifically, these affordances of YouTube captions and adjustable settings like font size and colour, which were utilized for their positive multimedia effects, were especially advantageous for learners to improve their writing accuracy. First, in terms of learners' attention, they drew the learners' attention to (new) target language input, helped them clearly notice their errors/gaps and made conscious comparisons between their own output and the target language input (as implied in the signalling principle and noticing the gap hypothesis 2.1.). Second, in terms of learners' comprehension, they helped the learners easily process more information in the working memory, increased their comprehension of the spoken language input and retained these information whenever required from the long-term memory (as implied in the multimedia principle 2.1.). Subsequently, learners could acquire or learn from the correct language used in some video and applied this new knowledge in their future writing. It should be mentioned that the language used in some video was taken as a model (i.e., which was supposed to be completely correct and error-free), especially the language of native speakers. In this study, the correct language, which was acquired by this group of learners and reflected in their L2 writing after the use of the proposed multimedia affordances of YouTube, included aspects related to the accuracy of word choice, grammatical structures/morphological forms, capitalization, punctuation and word spelling. In line with a study by Gass et al. (2019) asserting the positive role of captions, this study also concluded that enhanced captions could visually signal to ESL learners linguistically relevant information like word boundaries/word spelling, grammatical structures/morphological forms. This could help learners comprehend speech in the aural channel and draw their attention to the spoken language input presented to them in a multimedia learning environment, especially when YouTube was used as a tool to create such a learning environment. Also, findings of this study conform with those reported by Baltova (1999) that the potential effect of signalled bimodal captioning (i.e., audio and text are synchronized with videos in L2 as implied in the temporal contiguity principle, section 2.1.) could prompt an enhanced multisensory cognitive impact on listening recognition and long-term content retention for L2 learners. In this study, such positive multimedia effects clearly supported learners with their English writing. For example, owing to the enhanced captions effects, learners in this study could better recognize the new language (in terms of both new content and forms), clearly noticed their errors as they compared their error-prone language with the error-free language used in some video and ultimately recalled and employed the correct language in their future writing tasks. Some of the interesting examples about the kinds of errors (this group of learners would frequently make when they wrote in English) which were noticed and corrected with the help of enhanced captions of YouTube videos (i.e., by way of conscious comparison between their own output and target language input in these videos) were related to the right use of word choice (e.g., ∗take care about, ∗in Friday, ∗much calories), spelling (e.g., ∗pupulation, ∗dileshes), capitalization (e.g., the personal pronoun ∗i, ∗india), grammatical structures (e.g., ∗the India, ∗there's people, ∗she wants sleep), morphological forms (e.g., ∗I cannot stop eat, ∗the cat love the massage, ∗I'm agree), punctuations (e.g., …∗because., ∗Did you remember me., ∗So I am using technology…). Moreover, the multimedia principle is crucial to explain how this group of learners were able to process L2 more efficiently with these YouTube multimedia videos as the enhanced captioning facilitated processing language input where the cognitive processes of learners meld the incoming aural linguistic input with visual words (in this case captions) (Yang, 2020).

Taken together, the results of this study about the L2 writing accuracy development and the role and impact of the enhanced captions of YouTube videos (Table 1) were in line with recent studies like (Yang, 2020) about the cognitive advantages of YouTube captioned videos which revealed that L2 learners could process language more easily with enhanced captions, and thus the improvement of their overall language skills.

With regard to the second research question in this study, the quantitative findings (Table 2) clearly demonstrated with statistical evidence some advancement of the learners' L2 writing accuracy after the implementation of the proposed YouTube multimedia affordances (i.e., the enhanced captions) as a tool for creating and enhancing a multimedia learning environment for the learning and improvement of the ESL writing of this group of learners. The actual improvement of learners' writing accuracy, which was determined by the writing accuracy metrics used in this study (Table 2), involved four main areas, namely grammar, mechanics of writing, vocabulary and morphology.

The development of the learners' English writing in terms of the grammar and mechanics of writing was seen in the progress made by learners as indicated by the following writing accuracy measurements: *the number of errors per T-unit, number of errors per 100 words, ratio of error-free clauses* (Table 2). The results of this study about the development of these accuracy aspects of writing (i.e., the grammar and mechanics of writing) with the help of the YouTube multimedia affordances such as the enhanced captions supported previous research findings. Pratiwi (2011) and Anggraeni (2012) found that the use of YouTube multimedia affordances can help learners [in the process of writing] to select the right words and expressions, arrange words into sentences and sentences into paragraphs, write grammatically correct sentences, and correctly use the mechanics of writing (i.e., punctuation, word spelling). Also, Morris et al. (2016) found that, in terms of spelling of keywords, “students appreciated the chance to see how unfamiliar words were spelled. For example, learners reported that if the professor said a word we didn't understand, we'd go back and read the caption; there were many legal terms that we did not know of and the captions helped us learn how to spell them.”

The development of the learners' English writing in terms of vocabulary and morphology was seen in the progress made by learners as indicated by the following writing accuracy measurements: the *target-like use of vocabulary, percentage of target-like verbal morphology, target-like use of articles* (Table 2). These findings supported and added value to previous studies about the advantages of using captions for the improvement of vocabulary acquisition of L2 learners. Winke et al. (2010) noted that the use of captions induces deeper processing as they help learners to be more attentive to tasks, reinforce their acquisition of vocabulary through multiple modalities, and help them to figure out meaning through the unpacking of language chunks. From a cognitive perspective, Vanderplank (1988, 1990, as cited in Yang, 2020) found that the employment of nontypical input modes (i.e., video captioning) can facilitate processing language in chunks; this can be beneficial to overall L2 learning because the processing demands are reduced. Consequently, owing to the captioning effect which helps in chunk learning, “students can analyze and break down the aural input into meaningful constituent structures” (Winke et al., 2013).

Regarding the third research question in this study, generally, the findings (Table 2) about learners' English writing accuracy improvement post the integration of the enhanced captions of YouTube videos as a technique for the improvement of the learners' L2 writing accuracy revealed statistically significant correlations with the findings (Table 1) about the role and impact of the enhanced captions of YouTube videos on the learners' personal experience (Table 3). The results of this work supported findings of previous ESL correlational studies about the positive correlation between the development of the learners' L2 productive skills such as writing and the effective use of affordances of ICT multimedia tools like YouTube which can be employed for their multimedia learning effects in the learning environments. Izquierdo et al. (2015) noted that there is a positive influence on the learning behaviour of learners with increased motivation and better performance as a result of a strong and positive correlation between learning/improving L2 skills such as writing and the effective use of multimedia materials in a computer-enhanced language learning environment. Also, Alobaid (2020) found that there is a strong positive correlation between the writing fluency performance of L2 learners and their exposure to and high engagement with multi-media learning environments using ICT tools like YouTube, where text (i.e., closed caption) is optionally available for learners to be used along with speech while learning.

Finally, the qualitative results of this research (Table 4) supported and added value to the quantitative findings in this study. Also, they demonstrated more evidence about the positive role and impact of the enhanced captions of YouTube videos on the development of English writing accuracy of ESL learners. Such evidence was gathered according to the learners' experience and the benefits gained from the actual use and potential advantages of the enhanced captions of YouTube videos, which were used for the development of writing accuracy of ESL learners. For example, learners found that the use of the enhanced captions could make learning writing easier because it could optimize comprehension of YouTube videos content/topics, especially in the pre-writing stage. This suggested that enhanced captioning can be helpful to make YouTube videos content and language materials more comprehensible for learners before they start writing. In their study, Lwo and Chia-Tzu Lin (2012) also found that there is a positive effect of captions in multimedia L2 learning with respect to vocabulary acquisition and comprehension where learners performed better in learning English compared to those who did not have such captions.

Also, learners found that the enhanced captions attracted and focused their attention on their writing errors or gaps; this helped learners notice their errors/gaps, correct them and learn from their errors (especially their vocabulary, grammar and spelling errors) and ultimately improved their overall language use of English writing, i.e., through checking their writing, which was structured around the language used in some video, with the language used in some video, especially in the revision and proofreading stages of writing (given that the language used in some video is taken as a model, i.e., the language of native speakers). This showed the supportive role of enhanced captions for learners to notice their errors and learn from them especially when they revised and proofread their writing. In the same vein, learners found that the enhanced captions could attract and focus their attention on new language points (i.e., highlighted by the teacher or raised by the learners themselves) related to the target language use of grammar, vocabulary, spelling and punctuations; consequently, as implied by the signalling and multimedia principles, this could facilitate the retention of these newly learnt information and the overall process of learning and improving L2 writing. This showed that enhanced captions can be used to help drawing learners' attention and improve retention of newly learnt language and ultimately contributing to their overall L2 writing accuracy improvement. These results corroborated previous research that found captioning a video for L2 learners can improve their comprehension of, attention to, and memory for the video materials (Gernsbacher, 2015), enhance their written vocabulary (Parkhill & Davey, 2012) and increase their attention to the language learning materials (Steinfeld, 1998).

Finally, this study found that the use of the enhanced captions (by way of the proposed multimedia effects of boosting the learners' attention, comprehension, processing and retention of information for the improvement of their L2 writing accuracy) can create and enhance the intended multimedia learning environment for learners of different learning styles. This suggested the assistive role of enhanced captions to cater for different learning styles down the process of learning and improving the learners' L2 writing. This was in line with Morris et al. (2016) who reported that, in terms of comprehension, some students found the option to both hear and see language content of videos more consistent with their learning styles. These self-described “visual learners” treated captions as a core delivery method, not only supplement to the audio content. For instance, captions helped clarify misunderstandings/miscommunications and made the information much easier to learn for visual learners.

On the basis of the quantitative and qualitative findings in this study, YouTube multimedia affordances such as the enhanced captions seem to have multiple significant pedagogic and cognitive values which could contribute in multiple ways to the process of English writing improvement. This demonstrated the positive role and impact of multimedia affordances of YouTube as an ICT multimedia learning/teaching tool to create and enhance a multimedia learning environment for the development of ESL learners' writing accuracy.

### 5.1 Evaluation of learners' experience and pedagogical implications

This work explored the learners' experience and actual use of the YouTube affordances of enhanced captions, which were used for their multimedia learning effects, to determine their role and impact on the development of ESL writing accuracy.

While using these affordances for learning and improving English writing accuracy, learners were observed to be engaging with and benefiting from their multimedia learning effects. For example, these enhanced captions, which were adjusted in terms of font size and color for their signaling effects, could effectively attract the learners' attention to the on-screen text in a number of ways while learning in English writing classes. First, in terms of a better comprehension of the new target language input, learners used the enhanced captions technique to check out the right meaning of difficult/complex sentences in context, unfamiliar/problematic word meaning and searched for answers to questions mentioned in the videos (i.e., basically in the pre-writing stage). Second, in terms of the learners' writing errors/gaps, the enhanced captions were of great benefit to get the learners' attention to and retention of important information such as the unfamiliar or problematic word choice/spelling or grammatical structure mentioned in some video (i.e., basically in the revision and proofreading stages of writing). Third, based on the premises of the multimedia and signaling principles stated earlier (Mayer, 2014), the benefits of presenting information with the signaled text included (in this case YouTube on-screen enhanced captions) not only helped learners clearly notice their errors/gaps in their writing (and automatically correct them) but also helped them integrate (and later on recall) the new language input into a coherent linguistic and mental model. Therefore, ESL instructors need to think of linking linguistic elements to some visual stimuli (i.e., enhanced captions) in order to help L2 learners better comprehend/process, notice, store and recall the (new) target language input. This may explain how the right implementation of the affordances of YouTube multimedia videos in this study, namely the captioning (i.e., used for its multimedia effects) and the adjustable settings of font size and color (i.e., used for their signaling effects) helped learners notice and correct their errors; subsequently, their writing accuracy moved up to a higher level as it can be seen in (Table 2). These observations about the participants' experience in this study regarding the role of captioning supported and added value to previous studies. Froehlich (1988) as cited in Yang (2020) stated that learners pay dedicated and synchronized audio-visual attention to L2 speech as they watch L2 videos with captions. Winke et al. (2010) explained that ESL learners use captions to increase their attention, improve processing, reinforce previous knowledge, and analyze the target language. In other words, learners employed captions as a crutch.

Seemingly, it can be argued that the improvement of learners' writing quality in this study was due to the lucidity and intelligibility of the target language input presented in these YouTube videos which was enhanced by means of these ICT multimedia affordances, i.e., the enhanced captions. As such these multimedia affordances were beneficial for the creation and enhancement of the multimedia learning environment for ESL learners of intermediate language proficiency level, i.e., helped them write more accurately over time.

With special reference to the learners' L2 proficiency level, despite the consensus among researchers who examined how L2 learners use and benefit from the multiple pedagogic advantages of captions, the mixed findings of previous studies can be somewhat confusing. While Lwo & Chia-Tzu Lin (2012) found that lower-proficiency learners benefited more than higher-proficiency learners in terms of comprehension when provided with L2 English captions, other researchers reported larger benefits for intermediate and advanced L2 users when compared to beginners (see Vanderplank, 1988; Neuman and Koskinen, 1992; Taylor, 2005; Perez et al., 2013; Alobaid, 2020). Researchers like Leveridge and Yang (2013) stated that more proficient English learners relied less on captions to process information. Taylor (2005) concluded that a greater reliance on captions seems to require attentional trade-offs as lower proficiency learners reported difficulty in simultaneously processing audio, visuals, and captions. Quite recently, Yang (2020) found that enhanced captioning [within a multimedia learning context] plays a more positive role on the intermediate-high and advanced German learners' motivation likely due to more efficient language processing. More specifically, the videos are able to provide linguistic integrity, which allows the language learners to focus their incidental attention more effectively. As for this study, the learners' proficiency level was estimated to be intermediate and above. Hence, the major findings of this work are in line with the most recent research findings mentioned above. As a rule of thumb, we suggest that perhaps it should be left to instructors to make a better decision *how much captioning is needed?* and *whether to include it or not in the first place?* “depending on the objectives of the lesson, learners' needs and level of L2 proficiency of learners” (Chai and Erlam, 2008). This can be done while observing in real learning contexts how learners interact with captions and possibly benefit from their potential multiple advantages.

### 5.2 Usability issues and caveats

#### 5.2.1 Cognitive overload

Mayer (2014) stated that multimedia learning occurs when the target language input comes through more than one channel (i.e., aural and visual). However, as L2 learners have limited capacity for processing information presented in a single format, they need to be selective and attend to a particular input in a particular modality. Otherwise, there will be a cognitive-processing overload (Sweller, 2005; Paas and Sweller, 2014). Although research in multimedia learning demonstrated many cognitive advantages for L2 learning, our deep concern is that captions can cause cognitive overload in the learners' working memories. This can happen “as the combination of both speech and text (which is the case in this work) may overwhelm learners' visual channels according to the dual-channel and limited-capacity assumptions of CTML (Mayer, 2009), i.e., extraneous information impedes efficient processing” (see Kozma, 1994; Lee et al., 2006; Mayer and Moreno, 2003, as cited in Alobaid 2020). Therefore, this research recommends a well-balanced strategy when using captions for ESL learning and teaching; they can be included only if and as much as needed without compromising the working memory resources of learners. Also, this study recommends what Matus (2018) suggested when targeting certain linguistic points in multimedia presentations, i.e., using various signalling strategies to reduce extraneous processing and attract learners' attention.

#### 5.2.2 Captions inhibiting effects

Froehlich (1988) observed that beginner L2 learners may simply focus on captions with little or no attention given to the L2 spoken language while watching captioned L2 videos. Danan (2016) suggested that captioning may be disturbing or turn into a crutch for L2 learners when their focus is on the onscreen text rather than listening. Chai and Erlam (2008) noted that while learners watch captioned videos, they perhaps give priority to reading over listening. As such this might inhibit them from processing and benefiting from the input supplied by the contextual clues and aural channel; for this reason, instructors are suggested to alternately use captioned and non-captioned videos according to the objectives of the lesson, learners' needs and their L2 proficiency level.

#### 5.2.3 Accuracy of auto-generated captions

There is a concern over the accuracy of auto-generated language of the captions on YouTube. This can happen as the system of the automatic captions is prone to errors as these captions are machine-generated, so the captions quality may vary. For example, auto-generated captions would probably misrepresent the spoken content due to mispronunciations, accents, dialects, background noise or when there are multi-syllable words. A potential problem would be that L2 learners might accidentally mis-learn or instil wrong language forms as they might take it for granted that such language is perfectly correct, and this can be reflected in their L2 writing. Therefore, instructors are advised to constantly preview captions and edit the incorrectly transcribed parts for their learners.

## 6 Limitations

The small sample size is a limitation in such small-scale studies; thus, this researcher used non-parametric rather than parametric statistical tests. Therefore, the results of this research should be treated with some caution although they seem to be statistically significant. For future studies, the author urges for a substantially larger number of participants as this will not only validate the results of this research but will also yield more robust results regarding the potential impacts of YouTube multimedia videos with captions on L2 learners. Moreover, these findings are culturally bound to Arabic learners of English in an English-speaking country, India. As it was indicated in similar studies like (Yang, 2020) that “other cultures and languages might prove to be considerably different, especially for languages which do not use Romanized characters”.

## 7 Conclusions

This work clearly demonstrated that ICT multimedia learning tools like YouTube as an online open-source learning platform with respect to its affordances of captions and their adjustable settings like font size and colour (i.e., enhanced captions of videos) can be efficiently used and recommended for the development of learners' L2 writing accuracy due to their positive and enhancing multimedia learning effects. Subsequently, if rightfully used, these YouTube affordances can make the process of learning and improving writing easy, effective and above all learners' writing performance more accurate.

## Declarations

### Author contribution statement

Azzam Alobaid: Conceived and designed the experiments; Performed the experiments; Analyzed and interpreted the data; Contributed reagents, materials, analysis tools or data; Wrote the paper.

### Funding statement

This research did not receive any specific grant from funding agencies in the public, commercial, or not-for-profit sectors.

### Data availability statement

Data will be made available on request.

### Declaration of interests statement

The authors declare no conflict of interest.

### Additional information

No additional information is available for this paper.

## Footnotes

1 “Cognition consists of a set of skills and brain functions through which individuals perceive their environment” (Drigas and Pappas, 2017).

2 Enhanced captions: this term is used in this work to refer to the affordances of video captions which can be enhanced through use of the adjustable settings of font size and colour while streaming YouTube educational videos.

3 This study was approved by the Academic Research Committee at the Department of Linguistics, School of Languages, Literature and Culture Studies, J.N.U. New Delhi, India. Also, the participants who have volunteered to be part of this research work have been informed in detail about the nature and procedures of conducting this research. The researcher has obtained the prior consents of each participant before conducting the research survey and writing tasks/tests.

4 The researcher in this study took the full role of English language instructor in these English writing classes.

5 All the IELTS standards and criteria (i.e., time-wise, material-wise, number of tasks, etc.) were entertained and taken into consideration while the author administered the tests in the class before and after the intervention. Also, the permission of the IELTS community was obtained from the British Council to use the test samples for the writing tasks used in this research. The reason for choosing this IELTS test (i.e., the legitimacy of using learners' IELTS scores as a progress criterion) is because of its overall reliability estimate over the four modules (speaking, listening, reading, writing) gives a high coefficient of 0.95 according to Feldt and Brennan (1989), and also there are positive correlations between IELTS scores and learners' academic performance/GPAs and thus its predictive power of learners' academic performance (Bellingham 1995; Cotton and Conrow, 1998; Feast, 2002, as cited in Hashemi and Daneshfar, 2018).

6 Hunt (1970) defined T-units as "the shortest units into which a piece of discourse can be cut without leaving any sentence fragments as residue”.

7 It should be noted here that the lower the learners' scores based on this metric, the more accurate the learners' use of lexis is (Skehan and Foster, 1997).
